# Protective effect of polysaccharides isolated from the seeds of Cuscuta chinensis Lam. on 5-fluorouracil-induced intestinal mucositis in mice

**DOI:** 10.1590/acb370204

**Published:** 2022-05-02

**Authors:** Yanzhao Ji, Weidong Zhou, Wei Tan, Zewei Chen, Hanqi Lu, Yanting You, Chunyang Tian, Xinghong Zhou, Lin Zhou, Ren Luo, Xiaoshan Zhao

**Affiliations:** 1 MD. Nephrology Department – Shanxi Bethune Hospital – Shanxi Academy of Medical Sciences – Tongji Shanxi Hospital – Third Hospital of Shanxi Medical University – Taiyuan, and Syndrome Laboratory of Integrated of Chinese and Western Medicine – School of Chinese Medicine – Southern Medical University – Guangzhou, China; 2 MD. Department of Traditional Chinese Medicine – Xinyu People’s Hospital – Xinyu, China.; 3 MD. Department of Traditional Chinese Medicine – Guangdong Geriatric Institute – Guangdong Provincial People’s Hospital– Guangdong Academy of Medical Sciences – Guangzhou, China.; 4 MD. Syndrome Laboratory of Integrated of Chinese and Western Medicine – School of Chinese Medicine – Southern Medical University – Guangzhou, China.; 5 MD. Endocrinology Department – Nanfang Hospital – Southern Medical University – Guangzhou, China.

**Keywords:** *Cuscuta chinensis* Lam, Polysaccharides, 5-Fluorouracil, Protective Agents, Mice

## Abstract

**Purpose:**

To evaluate the protective effect of Cuscuta chinensis Lam. polysaccharides (PCCL) on 5-fluorouracil-(5-FU)-induced intestinal mucositis (IM) in mice.

**Methods:**

PCCL was orally administered at a dose of 20 mg·kg^–1^ for 7 days and its protective effect on 5-FU-induced IM (5-FU, 50 mg·kg^–1^ for 5 days) was evaluated by monitoring changes in body weight, degree of diarrhea, levels of tissue inflammatory factors (tumor necrosis factor α, interleukin 6, and interleukin 1β levels), apoptosis rates, and the expression levels of caspase-3, Bax and Bcl-2.

**Results:**

The severity of mucosal injury (as reflected by body weight changes, degree of diarrhea, height of villi, and damage to crypts) was significantly attenuated by PCCL administration. PCCL also reduced the levels of tissue inflammatory factors, the apoptosis rate, and the expression of caspase-3 and Bax, and increased Bcl-2 expression.

**Conclusions:**

PCCL administration may be significantly protective against 5-FU-induced IM by inhibiting apoptosis and regulating the abnormal inflammation associated with it.

## Introduction

5-fluorouracil (5-FU) is a commonly used drug in clinical practice for the treatment of gastrointestinal cancers^1^. It exerts its cytotoxic effects by blocking the conversion of uracil to thymine and so affecting the synthesis and repair of DNA^2^. However, in addition to tumor cells, normal cells are also exposed to 5-FU so that their structure and functionality are damaged, disrupting the normal regulatory systems of the human body^3^. Intestinal mucositis (IM) is one of the major side effects of 5-FU. It has been reported that approximately 40% of cancer patients who receive 5-FU treatment for solid tumors suffer with IM^4^ and more than 60% of those undergoing high-dose 5-FU for stem cell transplantation display the clinical manifestations of IM^5^. As a major side effect, IM not only limits the efficacy of 5-FU in terms of cancer treatment, but it also affects the patient’s quality of life. The apoptotic inflammatory cytokines are considered to be key pathogenic elements in the pathogenesis of 5-FU-induced mucositis^6^^-^ 8. The normal balance between proapoptotic members of the family (such as Bcl-2-associated X protein, Bax) and antiapoptotic components (for example, B-cell lymphoma, Bcl-2) could be disrupted by the administration of mucotoxic therapies^9^. Apoptosis is believed to be an early central event of the pathogenesis. Chemotherapy-induced transcription-factor activation results in the production of proinflammatory cytokines such as tumor necrosis factor (TNF)-α, interleukin (IL)-1β and IL-6^10^^,^ 11, and subsequently aggravates the 5-FU-induced mucosal damage in the small intestine.

It has been suggested that specific agents of traditional medicines or foods could offer better treatment options for IM induced by 5-FU than the drugs currently used. *Cuscuta chinensis* Lam. has traditionally been used as a tonic to improve liver and kidney conditions and sexual function, as well as to prevent senescence and abortion. *Cuscuta chinensis* contains many chemical components, such as flavonoids, alkaloids, lignans, and glycosides, amongst others, which have been suggested to be effective in treating some diseases. A number of studies^12^^,^ 13 have been carried out on some of the chemicals^14^^,^ 15 present in *C. chinensis*. However, there has been limited research reported aimed at exploring the effects of the polysaccharides isolated from *C. chinensis* on 5-FU-induced IM. It has been confirmed that a polysaccharide from *Radix codonopsi* (Campanulaceae) and a polysaccharide from *Schisandrae chinensis fructus* (Magnoliaceae) could act against 5-FU-induced IM in a mouse model, via an anti-inflammatory effect. However, whether polysaccharides from the seeds of *C. chinensis* (PCCL) could attenuate 5-FU-induced small IM in a mouse model has not been confirmed.

## Methods

### Drugs and preparation of the polysaccharide extract

The 5-FU for injections was purchased from Shanghai Xudong Haipu Pharmaceutical Co., Ltd. (Shanghai, China). It was diluted in physiologic saline to a concentration of 5 mg·L^–1^ before use. *Cuscuta chinensis* Lam was provided by Guangdong Hexiang Pharmaceutical Co., Ltd. (Guangdong, China). The powdered dry *C. chinensis* Lam (200 g) was homogenized and extracted two times with 1,600 mL of distilled water for 4 h at 98 ~ 100 °C. The whole extract was filtered and centrifuged at 1000× g for 30 min at 4 °C. The supernatant was concentrated to 100 mL and precipitated by the addition of 95% ethanol in 1:4.3 ratio (v/v) at room temperature. After 24 h precipitation, the sample was centrifuged as described above, and the precipitate was dissolved in 100 mL of distilled water. This process was repeated three times. The final precipitate was then washed with Sevag reagent (isoamyl alcohol and chloroform in 1:4 ratio) and freeze-dried, which yielded the crude PCCL. The crude PCCL from starting crude materials was approximately 17.6% (w/w). Total sugar in the crude PCCL was 92.34% (by the phenol-sulfuric acid method using glucose as standard solution)^16^. The extracted powder was dissolved in sterilized distilled water and filtered (0.22-μm-mesh filter) before being administered to experimental animals.

### High performance liquid chromatography (HPLC) to determine PCCL

Prior to HPLC analysis, a mixture of 1 mL 4 mol·L^–1^ trifluoroacetic acid and 1 mL 1 g·L^–1^ PCCL was hydrolyzed at 110 °C for 2 h and then cooled to room temperature. The solution was evaporated at 70 °C and dried. The residual trifluoroacetic acid was removed by adding methanol three times. After drying, 1 mL water was used to dissolve the solution. NaOH (200 μL; 0.3 mol·L^–1^) and 1-phenyl-3-methyl-5-pyrazolone (PMP) methanol solution (200 μL; 0.5 mol·L^–1^) were added to 200 μL of the solution. Then it was placed in a water bath at 70 °C for 30 min, cooled to room temperature, and 200 μL 0.3 mol·L^–1^ HCl solution added, followed by 1.2 mL water and 2 mL chloroform. The mixture was vortexed for 60 s and centrifuged (1500× g; 5 min). The supernatants were filtered through a 0.45 μm membrane.

The HPLC analyses were performed on a Luna C18 (Phenomenex) column. The mobile phase was 0.1 mol·L^–1^ potassium dihydrogen phosphate buffer solution (pH 6.8): acetonitrile (82:18), at a flow rate of 1.0 mL·min^–1^ with a detection wavelength of 254 nm. The injection volume was 5 μL and the running temperature was 30 °C.

### Animals and treatment

Seven-week-old male Specific Pathogen Free (SPF) grade C57BL/6 mice, weighing 18–22 g were provided by the Guangdong Medical Lab Animal Center (Guangdong, China). All mice were allowed to adapt to their diet and environment for 7 days before experimentation. They were kept in cages with free access to food and water, and were housed under a 12-h light/dark cycle at 22 ± 1 °C. All procedures were approved by the Standards for Animal Ethics in Guangzhou Institute of Sport Science^17^. Animals were randomly divided into three groups (n = 10 per group): control (PBS), 5-FU (5-FU), and PCCL (5-FU+PCCL; 20 mg·kg^–1^). The same volume of PBS was given to control animals as of the polysaccharide extract to PCCL-treated animals. 5-FU was injected intraperitoneally once daily at a dose of 50 mg·kg^–1^ for 5 days (days 2 to 7). Treatment groups had doses of PCCL administered by intragastric gavage 2 days before 5-FU injection began and twice daily for 7 days (days 0 to 7).

### Diarrhea and weight measurement

Diarrhea score and body weight were recorded daily from day 0. The severity of diarrhea was scored using the following scale: 0, normal (normal stool consistency); 1, slight (loose stools); 2, moderate (overt diarrhea with perianal soiling); and 3, severe (severe/bloody diarrhea with substantial tail soiling). Body weight change was expressed as a percentage of body weight: body weight at day n × 100 / body weight at day 0.

### Histopathology

For 12 h after the last administration, animals were fasted. Then, they were anesthetized with 1% pentobarbital sodium, the abdominal cavity was opened and approximately 2 cm of tissue was removed, 3 cm from the beginning of the jejunum. The removed tissue was fixed and stained with hematoxylin and eosin (H&E; magnification of 400×) for histopathological evaluation. Morphological changes were assessed by measuring the length of the jejunum, the depth of crypts, and the ratio of the length of the jejunum to the depth of the crypts.

### TUNEL assay

The terminal deoxynucleotidyl transferase dUTP nick end labeling (TUNEL) assay using the In Situ Cell Death Detection Kit (Roche, Basel, Switzerland) was carried out according to the manufacturer’s instructions^18^. Paraffin-embedded tissue sections were washed in xylene (2 times for 15 min), followed by hydration with a series of 100%, 95%, 85%, and 75% ethanol solutions and ddH_2_O (2 times for 5 min each). Then, slides were incubated with proteinase K (30 min at 37 °C). After washing with PBS (3 times), the slides were incubated with 50 TUNEL reaction mixture (1 h, 37°C) in a humidified dark slide box. Finally, slides were washed with phosphate-buffered saline (PBS) (3 times for 5 min each). The ratio of apoptotic cells to healthy cells was determined under a fluorescence microscope by counting the positively stained cells in four randomly selected visual fields.

### Measurement of TNF-α, IL-6 and IL-1β

Jejunum tissues (50 mg) were homogenized in 2 mL of PBS and centrifuged (3,500 rpm; 15 min); the supernatant was collected. Cytokine (TNF-α, IL-6 and IL-1β) concentrations were determined using enzyme-linked immunosorbent assay (ELISA) kits purchased from R&D Systems (Minneapolis, MN, USA) by measuring the optical density at 540 nm^19^. Each experiment was performed in triplicate.

### Immunohistochemical staining

Paraffin-embedded tissue sections (4-μm thick) were deparaffinized and rehydrated through a series of polyxylene and graded alcohol solutions. Antigen retrieval was achieved in 0.01 mol of citrate buffer (pH 6.0). The buffer was put in a microwave oven for 20 min, and then incubated with 3% (v/v) H_2_O_2_ to block endogenous peroxidase activity. The slides were incubated overnight at 4 °C with primary rabbit monoclonal anticaspase-3 antibody (1:500; Asp175; Cell Signaling Technology, Beverly, MA, USA), rabbit monoclonal anti-BAX antibody (1:200; E63; Epitomics, Burlingame, CA, USA) and rabbit monoclonal anti-BCL2 (1:200; 50E3; Cell Signaling Technology, Beverly, MA, USA), which were diluted in primary antibody dilution buffer (P0103; Beyotime, China). Following incubation with horseradish peroxidase-conjugated goat-antirabbit immunoglobulin G (074-1506; KPL, Beijing, China) for 50 min at room temperature, the slides were treated with a peroxidase/DAB ChemMate DAKO EnVision detection kit (Dako Citomation, USA). After staining, six high-power fields (400×) were randomly selected for each slide and the average proportion of caspase-3-, Bcl-2- and Bax-positive cells in each field was counted using the True Color Multi-Functional Cell Image Analysis Management System (Image-Pro Plus; Media Cybernetics, Rockville, MD, USA). Finally, the differences relative to the control group were calculated. All experimental assessments were carried out blindly by a single investigator.

### Statistical analysis

Statistical analyses were performed using SPSS 22. 0 software. Metrological data using single factor analysis of variance were expressed as means ± standard deviation. Variance homogeneity tests were performed using the Levene’s test. The least significant difference method was used for comparing two groups when variance was homogeneous, while the Dunnett T3 Method was used when irregular. A value of p < 0. 05 was taken to indicate statistical significance.

## Results

### Monosaccharide composition of PCCL

**Figure 1:**
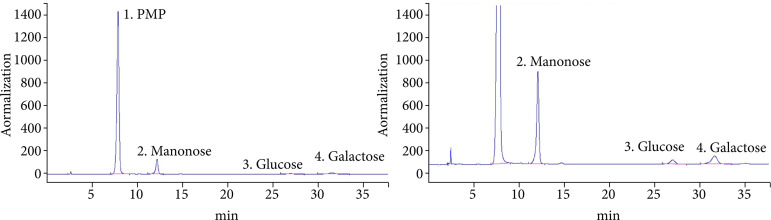
HPLC analysis of PCCL. Chemical structures of the monosaccharides comprising PCCL. Carbohydrates identified from reference to the peak numbers indicated on chromatograms. 1: PMP (PubChem CID: 4021); MW: 174.203 g·mol^–1^. MF: C_10_H_10_N_2_O; 2: Mannose (PubChem CID: 18950); MW: 180.156 g·mol^–1^. MF: C_6_H_12_O_6_; 3 Glucose (PubChem CID: 107526); MW: 180.156 g·mol^–1^. MF: C_6_H_12_O_6_; 4 Galactose (PubChem CID: 6036); MW: 180.156 g·mol^–1^ MF: C_6_H_12_O_6_.

The three characteristic peaks determined in the HPLC analysis of the hydrolysate of PCCL corresponded to the monosaccharides, mannose, glucose and galactose, as confirmed by comparing with mixed monosaccharide reference samples (Fig. 1); concentrations were 0.5668, 0.077 and 0.1678 mg·mL^–1^, respectively.

### Effect of PCCL on body weight loss and diarrhea score during 5-FU treatment

**Figure 2:**
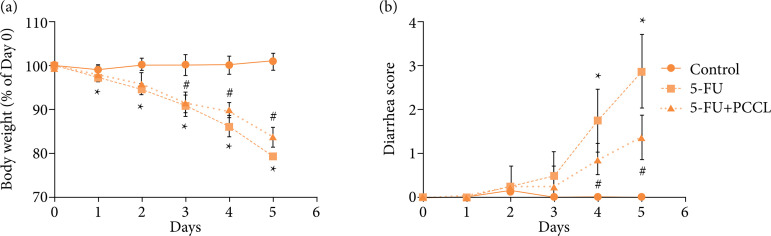
Effects of PCCL on changes in body weight and on diarrhea during 5-FU treatment. 5-FU (50 mg·kg^–1^) was injected intraperitoneally once daily, and PCCL (20 mg·kg^–1^) were administered orally twice daily for 6 days (days 0–5). **(a)** Body weight is shown as a percentage of initial body weight; **(b)** and the severity of diarrhea is scored using the four-grade scale (0 to 3). Data are the mean ± SEM of 8–10 mice.

The body weight of the 5-FU treated model group decreased significantly between day 1 and day 5 compared with the control group (p < 0.05; Fig. 2a). On day 5, the body weight percentage of the PCCL group was significantly higher than that of the model group (p < 0.05; Fig. 2a). 5-FU injection led to a rise in the diarrhea score on the 2nd day and a progressive increase until the 5th day of treatment, compared to the control group (p < 0.05; Fig. 2b). However, the diarrhea score of the PCCL group was well below that of the model group (p < 0.01; Fig. 2b).

**p < 0.05 vs. control (vehicle alone); #p < 0.05 vs. 5-FU.

### Effects of PCCL on the intestinal histopathological changes induced by 5-FU in the mouse small intestine

**Figure 3:**
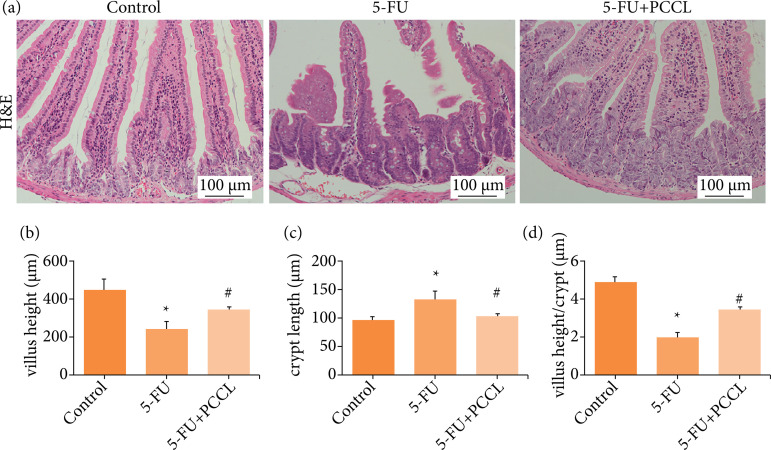
Effects of PCCL on the shortening of villus height and on crypt destruction induced by 5-FU in mouse small intestine. 5-FU (50 mg·kg^–1^) was injected intraperitoneally once daily, and PCCL (20 mg·kg^–1^) were administered orally twice daily for 6 days (days 0–5). **(a)** The jejunum was excised on day 6, sectioned, and stained with H&E (400×); **(b)** The height of villi; **(c)** the length of crypts; **(d)** and the villi height per crypt were measured. Data are the mean ± SEM of 8–10 mice.

Microscopic examination was used to analyze the intestinal histopathological changes in different experimental groups. In the control group, the intestinal villi were arranged in an orderly manner, the depths of the intestinal crypts were uniform, and the surface mucosal epithelia were continuous and intact. However, after continuous administration of 5-FU, the model group showed a difference in villi morphology with atrophy, deletion, and severe injury. Also, disorderly arrangement of the intestinal villi of mice was observed. The mucosal morphology of the PCCL-treated group was significantly improved, with an orderly arrangement of villi exhibited. Damage of the crypts was also repaired and the ratio, length of the jejunum villi/the depth of the crypt, was seen to increase (p < 0.05; Fig. 3).

*p < 0.05, vs. control (vehicle alone); #p < 0.05, vs. model (5-FU-treated).

### Effects of PCCL on TNF-α, IL-6 and IL-1β levels

**Figure 4:**
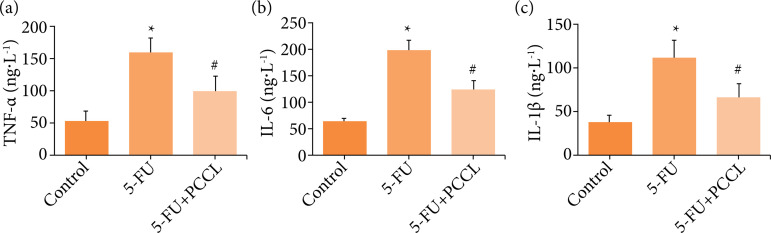
Effects of PCCL on TNF-α, IL-6, and IL-1β production in the jejunum. Cytokine (TNF-α, IL-6 and IL-1β)concentrations were determined using the appropriate enzyme-linked immunosorbent assay (ELISA) kit: **(a)** TNF-α levels;**(b)** IL-6 levels; **(c)** IL-1β levels. (ANOVA followed by Bonferroni’s test.) TNF-α: tumor necrosis factor α; IL-6: interleukin6; IL-1β: interleukin 1β; PCCL: *C. chinensis* polysaccharides; ANOVA, analysis of variance.

TNF-α is an early and important proinflammatory factor. It also promotes the production of other inflammatory factors such as IL-1 and IL-6, and plays an important role in the spread of inflammation^20^; therefore, TNF-α, IL-6 and IL-1β were used as indicators of the effects of PCCL on important inflammatory cytokines involved in the 5-FU-induced inflammatory response. 5-FU produced a significant elevation in inflammatory damage, shown as higher TNF-α, IL-6 and IL-1β concentrations compared to the control group (p < 0.05). Treatment with PCCL resulted in a marked down-regulation of TNF-α (p < 0.05; Fig. 4a), IL-6 (p < 0.05; Fig. 4b) and IL-1β (p < 0.05; Fig. 4c) expression. However, there was still a difference between the control and PCCL groups (p < 0.05).

*p < 0.05, vs. control; #p < 0.05, vs. 5-FU group.

### Effects of PCCL on 5-FU-induced apoptosis in the tissues of the small intestine

**Figure 5:**
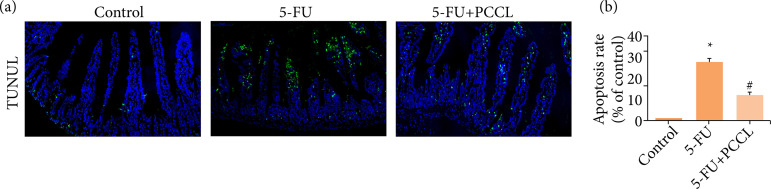
Effect of PCCL on apoptosis in intestinal crypts induced by 5-FU. 5-FU (50 mg·kg^–1^) was injected intraperitoneally, and PCCL (20 mg·kg^–1^) was administered orally twice, 30 min before and 8 h after 5-FU injection. **(a)** At the end of the experiment, the jejunum was excised 24 h after the final 5-FU injection, sectioned, and a TUNEL assay (400×) was performed; **(b)** The number of apoptotic cells was counted. Data are presented as the mean ± SEM of 8 mice.

To investigate whether the cellular protective effect of PCCL in small intestine tissue involved apoptosis, we firstly used TUNEL staining to detect cell apoptosis. Few TUNEL-positive cells were found in the control group, while a large number of TUNEL-positive cells were found in the 5-FU treated group (p < 0.05; 5-FU group vs. control group; Fig. 5) on day 1. Compared with the 5-FU-treated group, the administration of PCCL significantly reduced the number of apoptotic cells (p < 0.05; PCCL group vs. 5-FU group).

*p < 0.05, vs. control group; #p < 0.05, vs. 5-FU group.

### Effects of PCCL on Bcl-2, Bax, and caspase-3 levels

**Figure 6:**
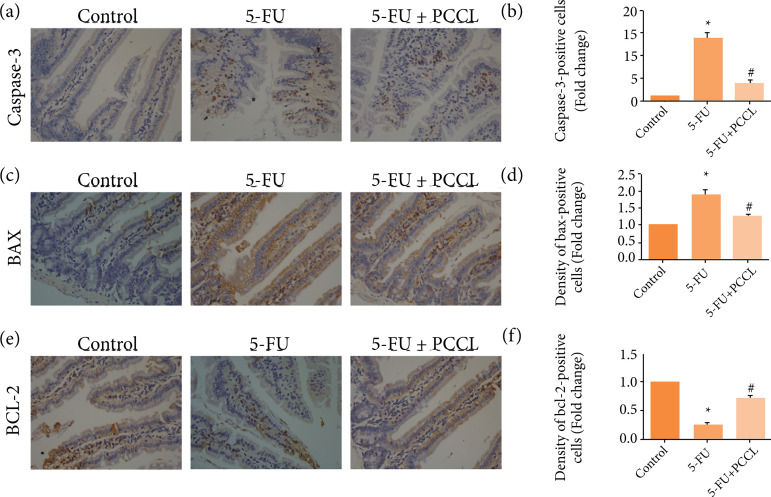
Immunohistochemistry of caspase-3, BAX, and BCL-2 in the jejunum Expression levels of caspase-3, BAX, and BCL-2 in the jejunum were assessed by immunofluorescent staining (400 ×). Positive cell numbers and staining intensities were assessed by the semi-quantitative method for evaluation as described in the Materials and Methods. **(a)** Images showing caspase-3 expression in each experimental group. **(b)** Data for caspase-3 expression (means ± SD). **(c)** Images showing BAX expression in each experimental group. **(d)** Data for BAX expression (means ± SD). **(e)** Images showing BCL-2expression in each experimental group. **(f)** Data for BCL-2 expression (means ± SD).

To elucidate further the protective mechanism of PCCL on 5-FU-induced apoptosis, proteins involved in apoptosis were examined using immunohistochemical technique. The relative expressions of caspase-3 and Bax were increased, while Bcl-2 expression was decreased in the 5-FU-treated group compared with the control group (p < 0.05; Fig. 6). However, in the PCCL group the 5-FU-triggered apoptosis was suppressed, with significantly lower expression of caspase-3 and Bax protein and higher expression of Bcl-2 (p < 0.05).

*p < 0.05, vs. control group; #p < 0.05, vs. 5-FU group.

## Discussion

5-FU is one of the most frequently prescribed drugs for gastrointestinal cancer patients^21^^,^ 22. However, continuous use of 5-FU usually leads to IM, resulting in a decline in the quality of life of patients and interruptions to treatment. Hence, a suitable drug to prevent this drug-induced IM is required with some urgency. Traditional Chinese medicines have attracted a lot of attention, as they have the advantage of a relatively low toxicity compared with modern chemotherapeutic agents. It is investigated that PCCL as a novel candidate derived from natural compounds for the treatment of IM induced by 5-FU in the present study. Diarrhea and body weight loss are reflective of the degree of IM. Here, typical pathogenic changes such as IM and morphological damage were observed, accompanied by a significant loss in body weight and severe diarrhea after 5-FU injection. Small intestine morphology was characterized by severe villus atrophy, flattening of the epithelium, and crypt destruction, which is known to be closely related to dysfunctionality of intestinal absorption. These findings are consistent with those of previously reported studies^23^^,^ 24, which have indicated that the 5-FU-induced IM model is valid.

PCCL has been demonstrated to have antiapoptotic effects on cardiomyocytes in aging rats^25^. It has also been shown to increase immunity, eliminate oxygen free radicals, and to exhibit anti-lipoperoxidation mechanisms in a senile mouse model^26^. In the present study, PCCL diminished the severity of intestinal damage caused by 5-FU, prevented the loss in body weight and the decrease in villus height, and reduced the occurrence of diarrhea. Therefore, PCCL might have beneficial effects on the IM induced by 5-FU.

It has been reported that apoptosis and inflammation are partially involved in the processes of 5-FU-induced IM. The mechanisms underlying the IM induced by chemotherapeutic drugs remain poorly understood. During the pathological process, a cascade of inflammatory pathways is triggered, and pro-inflammatory factors, such as TNF-α, IL-1β, and IL-6, are produced^27^. Then, proinflammatory cytokines exert directly damaging effects on mucosal target cells and amplify the inflammatory process via positive feedback loops, and so prolong tissue injury. As expected, the present study showed that 5-FU produced a significant elevation in inflammatory damage, with an increase in TNF-α, IL-6, and IL-1β levels. However, an amelioration of inflammatory cytokines was observed with PCCL treatment, meaning that PCCL may have anti-inflammatory effects on 5-FU-induced IM.

It is considered that apoptosis is a critical event in IM induced by 5-FU. Accordingly, significant apoptosis in intestinal crypts upon exposure to 5-FU was observed, which could have been triggered by proteolytic enzymes such as caspases. Caspases can be activated by two signaling pathways: the intrinsic pathway and the extrinsic pathway^28^^,^ 29. The intrinsic pathway is generally activated when proapoptotic proteins, such as Bax, are released from mitochondria^30^. The extrinsic pathway is initiated outside of cells via activation of death receptor signaling, e.g., by TNF-α. It has been previously reported that apoptosis of intestinal crypt cells induced by 5-FU is accompanied by increased proapoptotic Bax expression and decreased antiapoptotic Bcl-2 expression. Here, an upregulation of the rate of apoptosis was observed after 5-FU injection, accompanied by an alteration of caspase3, Bax, and Bcl-2 expression, indicating that the intrinsic apoptotic pathway had been activated.

Inflammatory cytokines can enhance apoptotic activity. TNF-α, IL-6, and IL-1β^37^ can facilitate cell apoptosis by altering the expression of Bax and Bcl-2 mediated by NF-kB protein. Members of the TNF family can activate caspase via the extrinsic apoptotic pathway. So, it is of interest to find here that a low dose of 5-FU (50 mg·kg^–1^) could lead to activation of both the intrinsic and extrinsic apoptotic pathway, which is opposite to the finding of Kato *et al*.^31^. However, with respect to the PCCL-induced decrease in apoptotic indexes found here, the results indicate that the protective mechanism of PCCL involves inhibiting both of the apoptosis signaling pathways. It has been reported that crypt apoptosis and the expression of inflammatory cytokines are sequential events in the development of IM after exposure to 5-FU. Detailed investigations into the mechanisms by which PCCL reduces the rate of apoptosis and alters levels of inflammatory cytokines to protect against 5-FU-induced IM are required in mice.

## Conclusion

5-FU-induced IM in mice PCCL can prevent the intestinal mucosa from becoming inflamed and can prevent apoptosis, and thus they represent a putative therapeutic agent for the amelioration of 5-FU-induced IM. Further studies will be required to determine more precisely their mechanism of action and suitability for clinical use.
